# Draft Genome Sequences of 27 Staphylococcus aureus Strains and 3 *Staphylococcus* Species Strains Isolated from Bovine Intramammary Infections

**DOI:** 10.1128/MRA.00300-20

**Published:** 2020-05-07

**Authors:** Soyoun Park, Dongyun Jung, Simon Dufour, Jennifer Ronholm

**Affiliations:** a Faculty of Agricultural and Environmental Sciences, Macdonald Campus, McGill University, Sainte-Anne-de-Bellevue, Québec, Canada; b Faculté de Médecine Vétérinaire, Université de Montréal, Saint-Hyacinthe, Québec, Canada; c Regroupement FRQNT Op+lait, Saint-Hyacinthe, Québec, Canada; d Mastitis Network, Saint-Hyacinthe, Québec, Canada; University of Maryland School of Medicine

## Abstract

Staphylococcus aureus is one of the most common etiological agents responsible for contagious bovine mastitis. Here, we report the draft whole-genome sequences, with annotations, of 27 S. aureus strains and 3 *Staphylococcus* species strains that were isolated from Holstein cows with intramammary infection in Canada.

## ANNOUNCEMENT

Staphylococcus aureus is a major pathogen that is responsible for both clinical and subclinical contagious mastitis in dairy cows. Dairy cows with active intramammary infection often have milk yield reductions. Persistent intramammary infections are a common cause of culling in dairy herds, which results in economic losses for the dairy industry (1). Although S. aureus-associated bovine mastitis cases have decreased since the implementation of mastitis control programs, S. aureus remains a challenge for dairy farmers and veterinarians (2, 3). In bovine mastitis control programs, prevention is the best strategy for reducing the burdens in the dairy industry. Although thousands of S. aureus isolates have been sequenced and reported, relatively few are from bovine intramammary infections. The availability of S. aureus isolates from intramammary infections will facilitate understanding of the molecular basis of pathogenic S. aureus characteristics associated with bovine mastitis. The Mastitis Network maintains a culture collection of mastitis isolates from Canada (4); each strain sequenced in this project was obtained from that collection.

Here, we present the draft genome sequences of 27 S. aureus isolates and 3 *Staphylococcus* sp. isolates from bovine intramammary infections in Canada. The isolates were initially identified at the species level using matrix-assisted laser desorption ionization–time of flight (MALDI) mass spectrometry, as described previously (5). Each isolate was cultivated from the –80°C stock on a tryptic soy agar plate, which was incubated overnight at 37°C. A single well-isolated colony was used to inoculate tryptic soy broth, which was incubated overnight at 37°C with agitation at 200 rpm. A 1.5-ml aliquot of the liquid culture was used for DNA extraction with the DNAzol reagent (Invitrogen) and lysostaphin (Sigma-Aldrich) according to the manufacturers’ instructions. Briefly, sequencing libraries were prepared as paired-end libraries with the Nextera Flex DNA library preparation kit (Illumina, San Diego, CA) and Nextera DNA CD indexes (96 indexes, 96 samples), and the libraries were sequenced on a MiSeq benchtop sequencer (Illumina) for 301 cycles in each direction. The reads were assembled *de novo* into high-quality draft genomes with ProkaryoteAssembly version 0.1.6 (https://github.com/bfssi-forest-dussault/ProkaryoteAssembly). Default parameters were used throughout, with the exception of the trimming step, for which the command trimq=20 2> {stats_out} was used to trim low-quality sequences with a Q score of <20. This assembly resulted in nonoverlapping contiguous sequences being generated for each genome (Table 1). Gene predictions and annotations were performed using the National Center for Biotechnology Information (NCBI) Prokaryotic Genome Annotation Pipeline (PGAP).

**TABLE 1:** Sequencing and annotation results for S. aureus and *Staphylococcus* sp. strains isolated from bovine intramammary infections

Strain identification no.	BioSample accession no.	GenBank accession no.	Draft genome size (bp)	Coverage (×)	GC content (%)	No. of contigs	No. of CDSs^*a*^	No. of RNAs
10508732	SAMN14230752	JAANCH000000000	2,735,287	141	32.72	14	2,668	65
41103371	SAMN14230753	JAANCG000000000	2,768,484	122	32.66	18	2,724	62
22200587	SAMN14230754	JAANCF000000000	2,723,469	144	32.72	16	2,653	62
30108394	SAMN14230755	JAANCE000000000	2,722,960	122	32.64	16	2,660	61
22516824	SAMN14230756	JAANCD000000000	2,721,244	159	32.73	14	2,653	64
31713214	SAMN14230757	JAANCC000000000	2,734,685	129	32.72	17	2,669	63
11105244	SAMN14230758	JAANCB000000000	2,728,884	132	32.73	15	2,653	64
32200324	SAMN14230759	JAANCA000000000	2,777,395	126	32.76	21	2,729	68
41704653	SAMN14230760	JAANBZ000000000	2,722,996	119	32.64	18	2,661	61
41302682	SAMN14230761	JAANBY000000000	2,685,527	69	32.68	19	2,614	61
30600096	SAMN14230762	JAANBX000000000	2,723,209	44	32.67	20	2,675	57
41012475	SAMN14230763	JAANBW000000000	2,732,002	43	32.72	34	2,662	65
10602379^*b*^	SAMN14230764	JAANBV000000000	2,493,911	68	35.77	25	2,445	66
31100823	SAMN14230765	JAANBU000000000	2,724,902	94	32.73	13	2,651	65
10400326	SAMN14230766	JAANBT000000000	2,640,591	111	32.61	22	2,562	58
32800326	SAMN14230767	JAANBS000000000	2,730,521	109	32.72	17	2,662	63
30704176	SAMN14230768	JAANBR000000000	2,691,107	96	32.67	18	2,621	60
11511212^*b*^	SAMN14230769	JAANBQ000000000	2,295,954	106	36.62	17	2,188	64
30500174	SAMN14230770	JAANBP000000000	2,726,086	82	32.69	19	2,670	60
10303344	SAMN14230771	JAANBO000000000	2,732,341	159	32.73	13	2,663	64
30300330	SAMN14230772	JAANBN000000000	2,736,609	103	32.72	21	2,671	64
21608872	SAMN14230773	JAANBM000000000	2,763,019	121	32.64	19	2,719	61
31500081	SAMN14230774	JAANBL000000000	2,736,124	161	32.71	15	2,669	59
11700739	SAMN14230775	JAANBK000000000	2,684,650	81	32.68	16	2,615	60
11007852^*b*^	SAMN14230776	JAANBJ000000000	2,457,092	134	35.65	42	2,389	65
41000044	SAMN14230777	JAANBI000000000	2,727,238	70	32.67	21	2,681	59
51000033	SAMN14230778	JAANBH000000000	2,683,716	154	32.64	21	2,619	52
31000024	SAMN14230779	JAANBG000000000	2,681,849	126	32.67	16	2,613	60
21000024	SAMN14230780	JAANBF000000000	2,729,466	61	32.71	22	2,666	62
31210331	SAMN14230781	JAANBE000000000	2,685,524	39	32.66	20	2,618	47

a CDSs, coding sequences.

b *Staphylococcus* species strain.

### Data availability.

These nucleotide sequences have been deposited in DDBJ/ENA/GenBank under the accession numbers provided in Table 1. The raw sequence reads have been deposited in the NCBI Sequence Read Archive under BioProject accession number PRJNA609123.
